# Mixing of porpoise ecotypes in southwestern UK waters revealed by genetic profiling

**DOI:** 10.1098/rsos.160992

**Published:** 2017-03-01

**Authors:** Michaël C. Fontaine, Oliver Thatcher, Nicolas Ray, Sylvain Piry, Andrew Brownlow, Nicholas J. Davison, Paul Jepson, Rob Deaville, Simon J. Goodman

**Affiliations:** 1 Groningen Institute for Evolutionary Life Sciences (GELIFES), University of Groningen, PO Box 11103 CC, Groningen, The Netherlands; 2 School of Biology, Faculty of Biological Sciences, University of Leeds, Leeds LS2 9JT, UK; 3 Institute of Zoology, Zoological Society of London, London NW1 4RY, UK; 4 Department of Zoology, University of Cambridge, Cambridge CB2 3EJ, UK; 5 EnviroSPACE Laboratory, Institute for Environmental Sciences, University of Geneva, Carouge, Switzerland; 6 INRA, UMR CBGP, 34988 Montferrier-sur-Lez Cedex, France; 7 Scottish Marine Animal Stranding Scheme, SRUC Veterinary Services, Drummondhill, Stratherrick Road, Inverness IV2 4JZ, UK; 8 Animal and Plant Health Agency, Polwhele, Truro, Cornwall TR4 9AD, UK

**Keywords:** ecotype specialization, molecular ecology, continuous population, dispersal, climate change, admixture

## Abstract

Contact zones between ecotypes are windows for understanding how species may react to climate changes. Here, we analysed the fine-scale genetic and morphological variation in harbour porpoises (*Phocoena phocoena*) around the UK by genotyping 591 stranded animals at nine microsatellite loci. The data were integrated with a prior study to map at high resolution the contact zone between two previously identified ecotypes meeting in the northern Bay of Biscay. Clustering and spatial analyses revealed that UK porpoises are derived from two genetic pools with porpoises from the southwestern UK being genetically differentiated, and having larger body sizes compared to those of other UK areas. Southwestern UK porpoises showed admixed ancestry between southern and northern ecotypes with a contact zone extending from the northern Bay of Biscay to the Celtic Sea and Channel. Around the UK, ancestry blends from one genetic group to the other along a southwest--northeast axis, correlating with body size variation, consistent with previously reported morphological differences between the two ecotypes. We also detected isolation by distance among juveniles but not in adults, suggesting that stranded juveniles display reduced intergenerational dispersal. The fine-scale structure of this admixture zone raises the question of how it will respond to future climate change and provides a reference point for further study.

## 1. Introduction

Intraspecific differentiation in contiguous geographical areas due to vicariance or environmental barriers is common in nature [1]. However, in the marine environment, movements are typically unrestricted over vast distances for highly mobile species such as cetaceans. This raises the question of how populations become genetically and ecologically differentiated with eventual speciation [2]. Despite their high dispersal ability, some cetaceans show substantial population structure, sometimes over a small geographical scale, not necessarily associated with geographical distance [2–4]. In some cases, oceanographic processes and/or behavioural traits explain a high level of population differentiation [4–9]. Prey availability, prey choice, social structure and/or other factors such as habitat availability, predator and competition pressure can all be involved in driving the pattern and extent of dispersal [1,3]. Explaining dispersal thus revolves around deciphering which current and/or historical mechanism(s) contributed to genetic structuring in the absence of obvious dispersal barriers.

The harbour porpoise (*Phocoena phocoena*) is one of the smallest and most abundant coastal cetaceans, widely distributed in subpolar to temperate coastal waters of the Northern Hemisphere [2,10,11]. Allopatric distribution, as well as morphological and genetic differences have justified the recognition of three subspecies of harbour porpoises: *P. p. vomerina* in the North Pacific Ocean, *P. p. phocoena* in the North Atlantic Ocean and *P. p. relicta* in the Black Sea [2–4,12–16]. In the eastern North Atlantic and Black Sea, numerous studies [4–9,13,16–20] assessed the population genetic structure of harbour porpoises during the last 20 years (reviewed in [11]). However, only recently, porpoises from southern waters of the Northeast Atlantic off the coasts of Iberia and Mauritania have been proposed as belonging to a fourth subspecies, *P. p. meridionalis* [11,18]. These southern porpoises were already known to be distinct with respect to their unusually large body size, often exceeding 200 cm, compared with the 150 cm of harbour porpoises found further north in the Atlantic and in the Black Sea [14,21,22]. Such morphological differences are probably related to genetic and ecological differentiation [11,18]. Indeed, these meridional porpoises inhabit a distinct environment [23], relying on the upwelling-related trophic network [24–26], which contrasts with the predominantly shallow habitat and demersal feeding habits of porpoises from the European continental shelf (e.g. [27,28]). While previous studies showed that porpoises from southern Europe were genetically differentiated [4,15,16,20], the extent of their genetic divergence was revealed by sequencing one-third of the mitochondrial genome [18]. Porpoises from Iberian and Mauritanian waters formed two distinct lineages clustering in a same monophyletic mitochondrial group with a level of divergence between the porpoises from southern and northern Northeast Atlantic as large as the divergence between the porpoises from the Black Sea and those from European waters north of the Bay of Biscay [18]. Given this level of divergence and other evidence of morphological and ecological differentiation, Fontaine *et al.* [18] proposed that the southern porpoises from Iberia and Northwest Africa were a distinct ecotype and Evolutionary Significant Units [29] from the porpoises inhabiting the continental shelf from the north side of the Bay of Biscay to the subarctic waters of Norway and Iceland. As such, the authors suggested that porpoises from Iberia and Mauritania should be raised to the level of subspecies, at the same taxonomic level as the porpoises from the Black Sea.

Coalescent-based reconstruction of the evolutionary history of the harbour porpoise populations showed that these three ecotypes in the Atlantic and Black Sea resulted from an initial split between the North Atlantic and Mediterranean porpoises, with the colonization of the Mediterranean Sea during the last Ice Age [11,18]. This event was followed by a split of the Mediterranean population into eastern and western groups from which descended the Black Sea population on one side [30] and the Iberian and Mauritanian populations on the other side. Finally, the Iberian population came back into contact with the northern continental shelf ecotype most probably during the Little Ice Age (*ca* 600 years ago), establishing a contact zone on the northern side of the Bay of Biscay, with predominantly northward gene flow [11,18,20,31]. However, the fine-scale spatial genetic structure of this admixture zone and the limits of its spatial distribution are still poorly understood. Previous studies had restricted sampling on the northern side of the Bay of Biscay, and in particular, there has been limited coverage of porpoises from around the UK.

**Figure 1.**
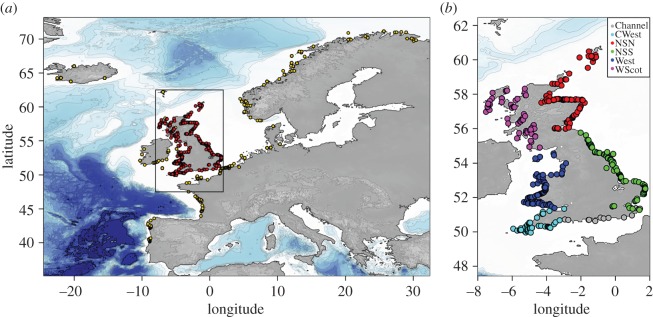
Geographical locations of the harbour porpoises sampling based on GPS coordinates or reported discovery location. (*a*) Global overview of the individuals considered in this study including the genotyped individuals from UK waters (red points) and the Northeast Atlantic individuals from Fontaine *et al*. [4] (yellow dots). (*b*) Locations of the UK samples have been subdivided into six regions around the UK and colour-coded accordingly for regional analyses.

In this study, we analysed the genetic structure of harbour porpoises around the UK using a dense sampling of 591 stranded animals (figure 1; electronic supplementary material , figures S1–S3) spanning a decade from 1990 to 2002 (electronic supplementary material, figure S4). We placed this ‘local’ genetic assessment within the global genetic structure of the harbour porpoises in the Northeast Atlantic by combining the UK dataset with previous data from Fontaine *et al.* [4]. We tested whether animals stranded around the UK show any evidence of mixed genetic ancestry from distinct genetic pools and morphological differentiation in terms of relative body size. Given the proximity of the Biscay admixture zone [4,18,20,31], porpoises in the southwestern part of the UK might be expected to show evidence of such mixed ancestry and could have larger body sizes, closer to Iberian porpoises. We also showed previously that gene flow and individual dispersal was restricted in space on the continental shelf north of the Bay of Biscay [4], creating a pattern of isolation by distance (IBD) [32,33]. Here, we tested whether such IBD exists around the UK and whether it differed between sex and age classes. Understanding the physical and ecological factors which influence the distribution of different ecotypes is central to understanding how this key pelagic predator may react to future climate change, and its subsequent impacts on Northeast Atlantic ecosystem [34].

## 2. Material and methods

### 2.1. Sampling

Tissue samples collected between 1990 and 2002 for 592 stranded or by-caught porpoises from the United Kingdom Cetacean Strandings Project (http://ukstrandings.org/) archives were provided by P. Jepson (Institute of Zoology, Zoological Society of London) and R. Reid (Scottish Marine Animal Stranding Scheme, SRUC Veterinary Services, Inverness). Data on individual sex, body size, weight, age (based on dental growth layer) and associated temporal, geographical and life-history data were collected following standardized detailed systematic post-mortem examination and sample collection protocols [35,36]. The distribution of the sampling in space, time and per category is shown in figure 1, table 1; electronic supplementary material, figures S1–S4. All maps in this study were generated in R statistical environment v. 3.2.4 [37] using the Marmap v. 0.9.5 package [38] and the ETOPO1 dataset available on the US National Geophysical Data Centre (NGDC) [39]. The sampling along the UK coast was subdivided into six zones (figure 1*b*) which correspond to the main distinct maritime areas around the UK and the principal stranding zones used by the United Kingdom Cetacean Strandings Network [40,41]. These distinct regions include the Channel, the Celtic Sea on the southwest coast (CWest), the North Sea North (NSN), the North Sea South (NSS), the West coast (West) and West coast of Scotland (WScot).

**Table 1.** Sampling distribution stratified by sex and age class. (n.a., not available.)

	females	males	n.a.	total
adult	86	108	1	195
juvenile	126	115	2	243
neonate	35	38	—	73
n.a.	38	41	2	81
total	285	302	5	592

### 2.2. Environmental data

Data on habitat characteristics across the study range with respect to salinity and sea surface temperature were taken from the National Oceanographic Data Centre (NODC) World Ocean Atlas (WOA01) [42]. Bathymetric data were extracted from the ETOPO2 dataset (NGDC) [39] and data on surface chlorophyll concentration were taken from the NASA Sea-viewing Wide Field-of-view Sensor database (SeaWIFS) [43]. To compare local habitat characteristics where harbour porpoises were living before dying, we calculated the mean value (±s.d.) of each variable within a radius arbitrarily set at 50 km around each sampling locality using the Spatial Analyst extension in ArcGIS™ v. 8.2 (ESRI®). This threshold distance was judged as a representative view of the environmental conditions where the porpoises were swimming before dying and stranding.

### 2.3. DNA extraction and microsatellite genotyping

Genomic DNA was extracted from skin or muscle sample using a standard phenol–chloroform protocol. Individuals were screened at 10 microsatellite loci used previously in harbour porpoises (Igf-1, 417/418, 415/416, GT011, GT136, GT015, EV94, EV104, GATA053, TAA031) (see [17]; electronic supplementary material, table S1). PCR reactions were carried out in 10 µl volumes overlaid with 10 µl of mineral oil using 1 µl of template DNA (approx. 10–50 ng µl^−1^); 1× PCR buffer with 1.5 mM MgCl_2_ (or 2.5 mM for loci GT015 and GT011, 2 mM for locus Igf-1), 0.23 U Amplitaq DNA polymerase (Perkin Elmer), 0.8 mM of each primer and 0.1 mM of each dNTPs except for dCTP which was 0.01 mM. PCR products were labelled during the PCR by direct incorporation of less than 1 µCi ^32^P-dCTP (0.09 mM). The PCR cycle programme for EV104, EV96 and EV94 was: 1× (95°C for 3 min); 7× (93°C for 1 min, 48°C for 1 min, 72°C for 50 s); 25× (90°C for 45 s, second annealing temperature for 1 min (electronic supplementary material, table S1), 73°C for 1 min) and a final extension (72°C for 15 min). We used the following PCR cycle programme for all other loci: 1× (3 min at 95°C); 35× (94°C for 1 min, annealing temperature for 30 s (electronic supplementary material, table S1), 72°C for 10 s) and a final extension (72°C for 15 min). PCR products from 96 individuals at a time were run on 6% denaturing polyacrylamide gels (Sequagel, National Diagnostics); visualization was performed by autoradiography using a Fujifilm BAS 2500 phosphor-imager. All genotypes retained for analysis were consistent across two or more genotypings, and all homozygotes were rerun at lower annealing temperature to check for potential allelic dropout after initial analysis for Hardy Weinberg equilibrium on genotypes from the first screen.

### 2.4. Data analysis

#### 2.4.1. Genetic diversity and differentiation around the UK

We estimated the proportion of missing data per locus and region using *poppr* packages [44] for the R statistical environment v. 3.2.4 [37]. Observed and expected heterozygosity (*H*_o_, *H*_e_), allelic richness (*Ra*) and inbreeding coefficient (*F*_IS_) [45] were calculated using *GENETIX* v. 4.05 [46] and *FSTAT* v. 2.9.3 [47]. These statistics were calculated per region (figure 1*b*). Per region *Ra* was computed based on a rarefaction procedure using the minimum sample size available across regions around the UK (*n* = 13). We conducted permutation tests (10^5^ permutations) in *FSTAT* to assess potential departures from Hardy–Weinberg (HW) equilibrium for each population. Confidence interval at 95% for the *F*_IS_ values were calculated using the *diveRsity* v. 1.9.89 [48] package for R [37].

We also investigated local patterns of genetic diversity by calculating *Ra* on a grid lattice of 2° where cells included at least two samples. We used a custom R-script to prepare the data, and *ADZE* 1.0 [49] to calculate *Ra* based on a standardized minimum sample size of two individuals. We plotted on a map an interpolated surface of *Ra* calculated using an inverse distance weighted procedure using *gstat* package for R [50].

Levels of differentiation in allelic frequencies between regional groups of porpoises were estimated using pairwise *F*_ST_ [45] values and 95% confidence intervals (CIs) calculated using the *diveRsity* [48] package for R. We considered *F*_ST_ comparisons as significant only if two conditions were met: the lower CI > 0, and *p* < 0.05 following a Bonferroni correction.

#### 2.4.2. Bayesian genetic clustering analyses

We analysed the genetic structure using a Bayesian model-based clustering method implemented in *STRUCTURE* v. 2.3.4 [51–53]. Since IBD has been previously observed north of the Bay of Biscay [4], we accounted for this by introducing into the Bayesian analysis a *prior* assumption that individuals found in the same area are likely to be more closely related to each other than individuals sampled from more distant locations. To implement this, we used the sampling location as a *prior* information in the Bayesian inference using the *Locprior admixture* model [53]. This model has better performance to detect existing genetic structure when the level of divergence is weak, yet without introducing biases towards detecting structure when it is not present [53]. Furthermore, we showed previously that this model provides a significant improvement to recover fine-scale genetic structure for the porpoises in the Northeast Atlantic by reducing the noise around estimates of individual admixture proportions [18].

*STRUCTURE* analyses were conducted by running a series of independent simulations with different numbers of simulated clusters (*K*), testing all values from 1 to 5. Each run used an admixture model with correlated allele frequencies, 1 × 10^6^ iterations after a burn-in of 1 × 10^5^ iterations. Ten replicates of each run were conducted to test for convergence of the Markov chain Monte Carlos. Results were post-processed using *CLUMPAK* [54] and custom R-scripts for comparing replicates to each other and identifying potential distinct clustering modes, estimating the change of likelihood and the best *K* value using the Evanno's method [55], and plotting the results.

**Figure 2.**
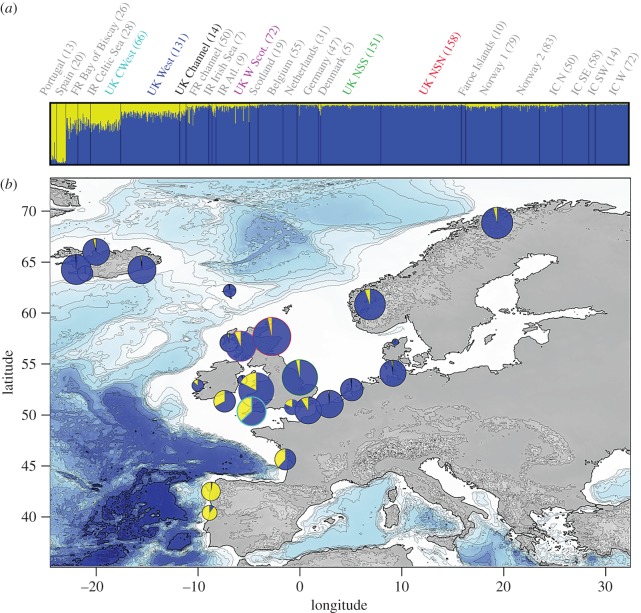
Genetic structure of harbour porpoises in the Northeast Atlantic with an emphasis on the genetic composition of the UK individuals. Admixture proportions estimated with structure at *K* = 2 (the most likely value; see the electronic supplementary material, figure S6) are shown at (*a*) an individual level (vertical lines on the barplot), with the individuals grouped according to localities (with the sample size between brackets) and sorted with increasing latitude. The averaged admixture proportions per geographical locality as defined on the barplot (*a*) and displayed on the map (*b*). Admixture proportions from structure analysis are based on the highest probability run (out of 10) at that value of *K* = 2.

We conducted this analysis at a local scale along the UK coasts, considering six zones which correspond to the main maritime areas around the UK (figure 1*b*). In addition, to put the local genetic structure into a broader context in the Northeast Atlantic, we combined the UK dataset with data previously obtained from Fontaine *et al.* [4,18]. The two datasets include, respectively, 9 and 10 microsatellite loci with an overlap for 6 loci and a total of 13 loci and a sample size of 592 new individuals from the UK coastlines and 676 from the other regions in the Northeast Atlantic [4,18] for a total of 1268 individuals (figure 1*a*). To calibrate allele sizes between the two studies, we genotyped 10 samples from the UK and 10 from Fontaine *et al.* [4,18] with the same protocol as described in [4,56] and aligned allele sizes between the datasets. We ran the *STRUCTURE* analysis on this enlarged dataset considering 25 sampled locations (figure 2), including the six zones along the UK coasts (figure 1*b*) and the 19 zones previously used in Fontaine *et al.* [4,18]

#### 2.4.3. Non-parametric multivariate analyses

Multivariate analyses of genetic data, such as principal component analysis (PCA), can provide a complementary view to the model-based Bayesian clustering approach [57,58], since these methods do not rely on any model assumption [59]. Therefore, we also analysed the genetic structure at the local scale around the UK using a spatial PCA (sPCA) [60], accounting for spatial autocorrelation, and aiming at displaying genetic variance with a spatial structure. We used a ‘global’ and ‘local’ test procedures based on Monte Carlo permutations (10^4^ permutations) to interpret the significance of the spatial principal components in the sPCA [60]. Following the definition of the sPCA, ‘global structure’ relates to patterns of spatial genetic structure, such as patches, clines, IBD and intermediates, whereas ‘local structure’ refers to strong differences between local neighbourhoods [60]. These analyses were conducted using the *adegenet 1.4-2* package [61] for R software [37].

#### 2.4.4. Isolation by distance analysis

Patterns of IBD may emerge if dispersal is spatially restricted at the scale of our study [32]. Under the hypothesis of IBD, genetic differentiation between individuals (estimated using the *â_r_* statistics analogous to *F*_ST_/(1 − *F*_ST_) between demes) is expected to increase with increasing geographical distance [33,62,63]. We calculated the regression coefficient (*b*) between genetic and geographical distance matrices between individuals and evaluated its significance with a Mantel Test (10^4^ permutations of geographical locations) using *SPAGEDI* 1.4 [64]. Instead of using a Euclidian distance that would poorly describe the actual geographical distance between pairs of individuals, we computed a marine geographical distance that accounts for the shortest path by sea between two individuals as described in Fontaine *et al.* [4]. To compute this marine geographical distance, we used a Least Cost Path algorithm using a modified version of *PATHMATRIX* [65], implemented in *C* for improved computational efficiency (available upon request to N. Ray).

We tested the occurrence of IBD first on all individuals around the UK. Then we tested whether IBD patterns differed among sex and age classes. IBD patterns could indeed differ among sexes and age classes if one of the classes (e.g. juveniles or females) disperses less than other classes (e.g. adults or males). We tested IBD in adults versus juveniles only, as sample sizes (table 1), spatial (electronic supplementary material, figure S1 and S2) and temporal distributions (electronic supplementary material, figure S3 and S4) were not sufficient to partition the data further and maintain satisfactory statistical power.

#### 2.4.5. Morphological analysis

Data on body length, age and sex were available for a large subset of the UK individuals (*n* = 336) included in the genetic analyses. As two porpoise ecotypes are present in the study area and are known to differ according to their body size [22], we investigated how body length varied as a function of the animal age and sex using a linear model. We were interested in the residual variation not accounted for by the age and sex and in particular its geographical component. Residual variation in body length was compared among the six UK geographical zones with an ANOVA in R [37] using log-transformation for body length and age. We also assessed the correlation between individual residual body size and individual admixture score derived from the *STRUCTURE* analysis.

## 3. Results

### 3.1. Genetic diversity and differentiation between regions around the UK

The proportion of missing data observed at the 10 microsatellite loci for the UK individuals ranged between 0.5% and 4.9% (electronic supplementary material, figure S5). All loci but EV104 showed less than 10% missing data in any of the six geographical regions around the UK (figure 1*b*; electronic supplementary material, figure S5). We excluded locus EV104 from further analyses as the proportion of missing data exceeded 10% in some regions (electronic supplementary material, figure S5) and potential null alleles have been recorded in other studies [4]. The genetic diversity (also known as expected heterozygosity, *H*_e_) of the remaining nine loci is shown in table 2, and ranges between 0.67 and 0.71 with an average value of 0.69 ± 0.01 across the six regions. The allelic richness per region ranged between 6.5 and 7.2 alleles for a standardized sample size of 13 individuals (the lowest sample size observed in the Channel area). Overall, none of the loci displayed any significant departure from HW and Linkage Equilibrium expectations.

**Table 2.** Genetic variation at the nine microsatellite loci per region and overall. (*N*, sample size; *n*_Al_, number of alleles; *Ra*, allelic richness for a standardized sample size of 13; *H*_e_ and *H*_o_, expected and observed heterozygosity; *F*_IS_, fixation index [95% CI obtained from 10^4^ bootstrap resampling].)

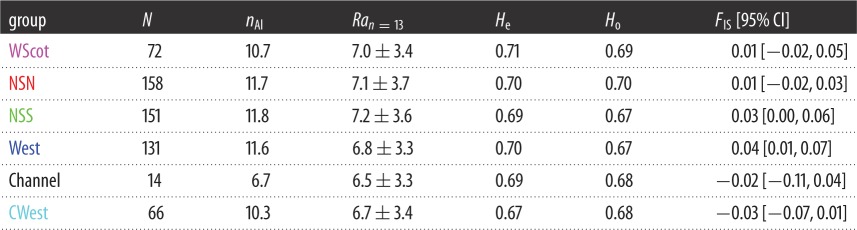

### 3.2. Genetic structure

Differences in allelic frequencies estimated using *F*_ST_ between porpoises from the six UK regions ranged between 0.0% and 1.3% (table 3). Only porpoises from the CWest group in the southwestern UK display consistently small but significant *F*_ST_ values when compared to porpoises from the five other geographical regions, indicating that porpoises from that area are differentiated from the others.

**Table 3.** *F*_ST_ value [95% CI estimated using 10^4^ bootstrap resampling] (below) and *p*-value estimated using 10^4^ permutations (above). (In italics are the pairwise comparisons that are statistically significant after a Bonferroni's correction at *α* = 0.05 and with a low 95% CI > 0.)

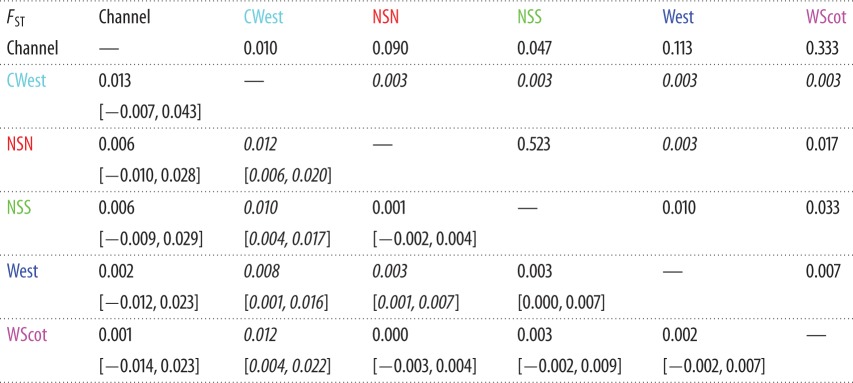

The individual-based Bayesian clustering analyses of *STRUCTURE* identified two clusters (*K*) as the best solution explaining the genetic structure of the harbour porpoises from the Northeast Atlantic, as suggested by the posterior probability of the data Ln (*P_K_*_=2|X_) = 1 for *K* = 2 and by the Evanno's method based on the rate of change of this posterior probability (figure 2; electronic supplementary material, figure S6*a*,*b*). The analyses conducted using the local dataset along the UK coastlines and at a global scale in the Northeast Atlantic by combining the UK dataset together with previous data of Fontaine *et al.* [4,18] (figure 1) depicted a very similar picture (figure 2; electronic supplementary material, figures S6 and S7), with a much clearer pattern observed when UK individuals were placed into the global genetic structure of the porpoises in the Northeast Atlantic (figure 2; electronic supplementary material, figure S6). These two analyses show that porpoises from the southwestern UK facing the Celtic Sea clearly have admixed ancestry from the northern (blue) and southern (yellow) ecotypes, similar to that previously identified for porpoises from French waters in the northern Bay of Biscay, and in Irish waters of the Celtic Sea [18]. These results were consistent across the 10 replicated runs conducted for each dataset (result not shown). The admixture zone between the two ecotypes is restricted to the northern part of the Bay of Biscay, and southern parts of the Celtic Sea and Channel. It includes porpoises found along the Atlantic coasts of France; the southern coasts of Ireland and northern coasts of Devon and Cornwall facing the Celtic Sea; and parts of the Channel. This is shown by the admixture proportions estimated at the individual level (figure 2*a*); as pie charts on the map showing the averaged admixture proportion per locality (figure 2*b*); and by colour-coding hybrid individuals which have less than 80% of their genome assigned to either of the two clusters (electronic supplementary material, figure S6*c*).

The newly analysed individuals from the UK coastlines thus allow for further refinement of the delimitation of this admixture zone. All the porpoises from the southwest coasts of UK (CWest) are part of this admixed zone between the two ecotypes, as are individuals from the west coasts of UK and the Channel. This admixed background quickly declines in porpoises further east into the Channel and along the UK coasts of the North Sea, as well as northwards along the coasts of Scotland (figure 2).

**Figure 3.**
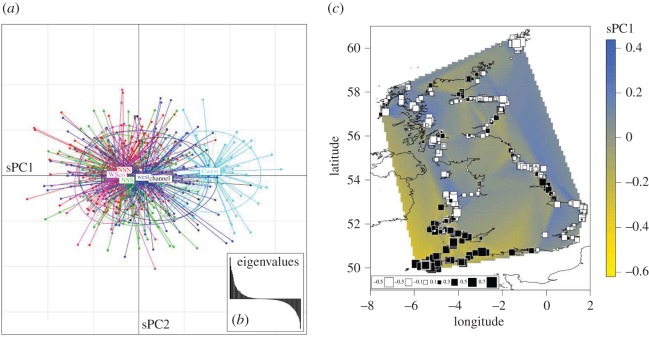
Spatial principal component analysis (sPCA) of the UK harbour porpoises. (*a*) The scores for each individual genotype are plotted for the first two sPCs, with colours indicating the discovery localities (figure 1*b*). (*b*) The inset provides the positive and negative eigenvalues. (*c*) Individual scores for the first component of the sPCA are displayed on the map using a size gradient of squares and a spatial interpolation surface.

The spatial principal component analysis (sPCA) conducted only on the UK porpoises provides a similar picture of the fine-scale genetic structure along the UK coastline (figure 3) and confirmed the results obtained by the Bayesian model-based clustering of *STRUCTURE* using a method that does not rely on any model assumptions. The Global test assessing the significance of positive sPCs showed that the first sPC is significant (*p* = 0.004) and supports the existence of a global genetic structure such as cline or clusters [60]. By contrast, the local test showed that none of the negative sPCs were significant (*p* = 0.598). Plotting the individual scores along the first two positive sPCs (figure 3*a*) showed that porpoises from the southwestern region of UK (CWest) depart from the others along the first sPC axis and that the genetic composition of British porpoises gradually changes along a southwest--northeast geographical axis (figure 3*a*). This spatial structure is also well depicted when plotting individual scores for the sPC1 on a map (figure 3*b*).

#### 3.2.1. Isolation by distance in the UK porpoises

We found significant IBD between the 591 porpoises sampled along the UK coasts, indicating that gene flow, and thus individual dispersal, is spatially restricted at that spatial scale (table 4). The IBD slope was similar between males and females, suggesting no evidence of one sex dispersing more than the other. When structuring by age-class, only the test performed on juveniles led to significant IBD, while the test conducted on the adults was not significantly different from zero. This suggests that the global IBD signal is primarily related to juveniles, while adults display, on average, a higher variance in dispersal.

**Table 4.** Isolation by distance conducted at individual levels between porpoises. (*N*, sample size; no. pairs, number of pairs considered in the analysis; *b*, regression slope; *p*-value (*b*_Obs_ > *b*_Exp_), *p*-value that the observed regression slope is higher than the simulated slope expected from 10^4^ permutations of the geographical distance matrix.)

	*n*	no. pairs	mean (max) distance (km)	*b*	*p-*value (*b*_Obs_ > *b*_Exp_)
overall	591	174 345	716.9 (1531.0)	4.48 × 10^−09^	*0.004*
adults	191	18 721	720.5 (1499.7)	1.41 × 10^−09^	0.322
juveniles	241	28 920	719.0 (1490.4)	5.67 × 10^−09^	*0.002*
females	285	40 470	722.6 (1499.7)	3.89 × 10^−09^	*0.041*
males	302	45 451	713.6 (1531.1)	4.26 × 10^−09^	0.051

#### 3.2.2. Morphological analyses of the UK porpoises

**Figure 4.**
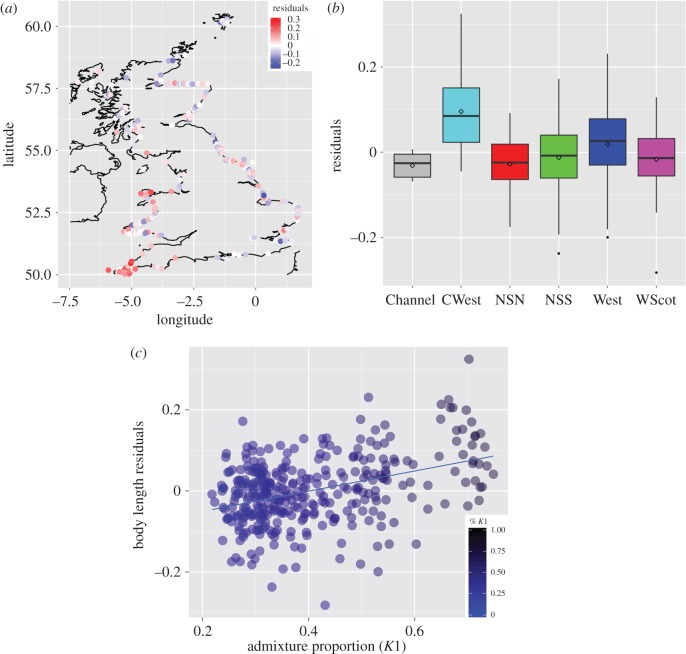
Geographical variation in the residuals from the linear model of the body-length values as a function of age and sex. (*a*) Residual values are shown on a map and (*b*) as boxplots per region. (*c*) The relationship between the individual residuals of body size with individual genetic admixture proportions (%*K1*) estimated in the Bayesian clustering analysis of structure (Pearson's *r* = 0.39, *p* = 8.3 × 10^−14^).

As previously reported [14], we found that both age and sex were significant predictors of the body length, explaining about 61% of the total variation (linear model, LM1: *F*_2,334_ = 261.1, *p* < 2.2 × 10^−16^, *n* = 336). We inspected the geographical variation in the residuals (figure 4*a*,*b*) and observed that porpoises from the southwestern (CWest) area as well as some porpoises from the west area of England displayed significantly larger body size compared to the others (one-way ANOVA, *F*_5_ = 15.53, *p* < 9.9 × 10^−14^ and *p* < 0.001 for all Tukey pairwise comparisons involving CWest; electronic supplementary material, table S2). We also observed a strong correlation between individual residuals of body size and individual genetic admixture proportions estimated by *STRUCTURE* (Pearson's *r* *=* 0.39, *p* = 8.3 × 10^−14^, figure 4*c*). Combining the genetic ancestry together with the age and sex in the linear model for predicting the body length increased significantly the total variance explained by the linear model up to 67% (LM2: *F*_3,333_ = 225.5, *p* < 2.2 × 10^−16^). This model with genetic ancestry offered a significantly better fit to the data compared to a model where it is not included (nested model comparison LM1 versus LM2: ANOVA *F*_1,333_ = 60.8, *p* < 8.2 × 10^−14^).

## 4. Discussion

Harbour porpoises in UK waters are part of a genetic continuum, characterized by a weak genetic structure, in which geographically proximate individuals are genetically more similar, a so-called IBD pattern [4,18]. However, porpoises stranded along the southwestern coasts of the UK, facing the Celtic Sea and the Atlantic side of the Channel, display significant genetic differentiation compared with those of other parts of the UK (figures 2 and 3). The genetic distinctiveness of the southwestern UK porpoises was shown independently by pairwise *F*_ST_ comparisons (table 3), Bayesian clustering analysis (figure 2; electronic supplementary material, figures S6 and S7) and sPCA (figure 3). Body sizes of southwestern porpoises are significantly larger compared with those of the rest of the UK (figure 4), being reminiscent of the large porpoises of the southern ecotype inhabiting coastal Atlantic waters of Iberia [18,22]. A significant correlation was found between body size and admixture proportion throughout the porpoise distribution around the UK. To our knowledge, this represents one of the largest assessments of body size variation in European porpoises to date. It shows that genetic differentiation correlates with the morphological differentiation observed between the northern and southern ecotypes, and suggests a potential genetic basis to traits with adaptive significance such as body size.

This pattern of genetic and morphological variation in UK waters is driven by the admixture between the two ecotypes previously identified in the Northeast Atlantic waters [18]: the southern ecotype known for their large body size [22] inhabits the upwelling waters off Iberia and Mauritania; and the northern ecotype that has smaller body size lives on the continental shelf north of the Bay of Biscay and spreads northwards up to the Arctic waters of Norway and Iceland (figure 2). The dense sampling along the UK coasts belongs to the same cohort (1990–2000) as those previously analysed in Fontaine *et al.* [4,18], and provides a refined picture of the global genetic structure, clearly showing for the first time the full delimitation of the admixture zone between the two ecotypes in the Northeast Atlantic. Our results show that the admixture zone is confined to the northern side of the Bay of Biscay and includes porpoises found along the coasts of France, Celtic Sea, southwestern UK, southern Irish Sea and the western side of the English Channel (figures 2 and 3; electronic supplementary material, figure S6*c*). The admixture proportions quickly decline along a southwest--northeast axis around the UK, blending towards pure individuals from the northern ecotype, with a coincident decrease in body size (figure 4).

**Figure 5.**
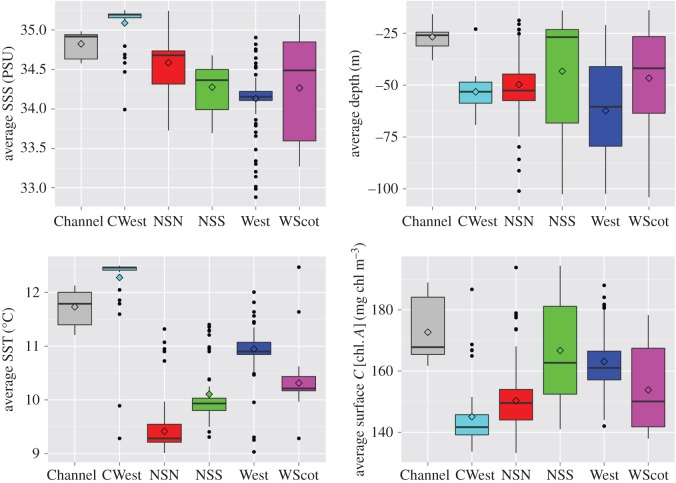
Boxplot describing the environment along the UK coastline within a 50 km radius surrounding stranded harbour porpoises. Annual sea surface salinity (SSS), temperature (SST), depth and sea surface chlorophyll concentration are shown.

While Iberian porpoises were already recognized as having larger body sizes compared with the northern ecotype [22], the new results show that the admixed porpoises found in the northern part of the Bay of Biscay are also relatively larger compared to the porpoises from the pure northern ecotype. These genetic and phenotypic differences strengthen the case that porpoises from each ecotype are part of demographically independent units, relying on distinct environments and different food resources. The local marine environment where the admixed porpoises from the southwestern UK (CWest area) were living before stranding showed substantial differences compared to other regions of UK with waters that are warmer, saltier and with slightly lower surface chlorophyll concentration on average (figure 5). From a biogeographic perspective, this area encompassing the Celtic Sea, the western English Channel and more generally the northern Bay of Biscay corresponds to a transition between two biogeographic marine zones (the Boereal–Lusitanean transition following [66]): the warm-temperate waters and cool-temperate waters [67]. The distribution of this admixture zone (figure 2; electronic supplementary material, figure S6*c*) in the northern part of the Bay of Biscay may just reflect a temporal snapshot, but could also be indicative of distinct habitat preferences of porpoises from the admixed zone and the southern ecotype compared to those living further north on the continental shelf. It may also be possible that some local adaptive processes are maintaining these two ecotypes as ecologically and demographically distinct. Testing these hypotheses would require a temporal study of the evolution of this admixed zone and should include a genome-wide perspective of the genetic differentiation to identify molecular evidence of ongoing natural selection.

Interestingly, porpoises from southwestern coasts facing the northern part of the Bay of Biscay displayed slightly lower genetic diversity compared to more northern porpoises (electronic supplementary material, figure S8). A previous genetic study reported a similar pattern at a larger scale in the Bay of Biscay together with a stronger IBD pattern than in the North Sea (see table 2 in Fontaine *et al.* [4] and table S8 in Fontaine *et al.* [18]). Such reduced genetic diversity in a zone of admixture might appear counter-intuitive at first glance, since we would usually expect an increase in genetic diversity when two distinct populations meet in a contact zone. However, we showed previously that the genetic diversity of the Iberian population is very low and does not have any private alleles relative to the northern continental shelf populations. Therefore, the reduction in diversity of the Biscay contact zone could arise through a combination of low genetic diversity of the southern ecotypes, and a high level of unidirectional gene flow from the Iberian population to the northern populations [4,18,31]. This results in a smaller effective population size and stronger IBD slope, which is inversely related to the neighbourhood size—the product of local effective density or population size and squared variance of the intergenerational dispersal distance [33,62,63].

The IBD observed around the UK was weak but highly significant, and consistent with patterns observed in other parts of the range [4]. When decomposing by age-class, we observed that the IBD pattern was only significant for juveniles but not adults. To assess the ecological significance of this requires some consideration of the statistical properties of the IBD test. An ideal test would compare IBD slopes between adults and juveniles and not just rely on the statistical significance of the test. However, the individual-based IBD test of Rousset has low power owing to the high variance inherent to Rousset's *a*_r_ estimator, which leads to an imprecise estimate of the slope [62,63]. The fact that the slope of the regression for adults is not different from 0 makes this slope comparison practically impossible, or would require another statistical framework, such as a Bayesian or likelihood approach. As described in Rousset [33,62,63], the strength of IBD and thus the level of genetic differentiation between individuals at a local scale in a continuous population can be quantified by its slope and is inversely proportional to the ‘neighbourhood size’. This ‘neighbourhood size’ can be described more precisely as the product of the local effective density *D* and the variance of intergenerational dispersal distance *σ*^2^. While local effective density or effective population size cannot explain the difference, we observed between juvenile and adult porpoises because they are part of the same population, the most biologically sound interpretation is that juveniles have a reduced intergenerational dispersal distance compared to adults. Intuitively, this would be expected if adults show some philopatry and faithfulness to particular breeding areas, as suggested in harbour porpoises, especially in females [68–71], and then disperse again the rest of the year (e.g. for foraging). Adults found stranded have thus more time and opportunity to disperse further away from their birthplace than juveniles. The intergenerational dispersal distance and especially its variance component should thus be much higher in adults than in juveniles, leading to a reduced ability to detect any IBD in adults but not in juveniles. This result is interesting, since it highlights how an indirect genetic approach based on uncontrolled sampling of stranded individuals can be informative for intergenerational dispersal behaviour in a species, such as the harbour porpoises, which has a continuous distribution and no geographical constraints on dispersal.

## 5. Conclusion

The evidence of an admixed contact zone between northern and southern porpoise ecotypes, occurring in the northern Bay of Biscay has been identified recently [18], but the present study fills an important gap along the UK coastlines that existed in the sampling distribution of previous studies. Using a very dense sampling around the UK, we were able to map with high resolution the geographical delimitation of the admixture zone, showing that porpoises from the northern Bay of Biscay, Celtic Sea and southwestern UK were genetically admixed. We showed also that the genetic admixture proportions were correlated with the body size of the porpoises, a discriminant morphological feature of the two ecotypes. This study revealed that not only are porpoises from the southern ecotype larger compared to the northern ecotype, but so are the admixed porpoises. This suggests that the body size of porpoises may have some genetic determination and also reinforces the idea that the two ecotypes display distinct feeding ecology, with potentially also distinct behavioural ecology and habitat preferences.

The current delimitation of the admixture zone raises the question of what environmental and ecological factors determine the distributions of the ecotypes, extent of the contact zone and whether the distributions are stable or dynamic. Previous work has shown that the structure and distribution of harbour porpoise populations has been influenced by changes in oceanographic conditions which affect food resources [4,18]. Therefore, the location and extent of the Biscay admixture zone is likely to be similarly dynamic and sensitive to past and future changes in climate which influence shifts in oceanographic and ecological conditions. For instance, warming waters may see a northward expansion of the southern ecotype, which would be detectable by a shift in the extent of the admixture zone around the southwestern UK. The data presented here represent samples spanning an approximate 12 year window during 1990–2002. In that time window, stranding records have been relatively constant between 1990 and 1997 and increased significantly after 2000 in the Bay of Biscay, Channel and southern North Sea [72], consistently with reported population movements [73]. Future studies, making use of the now extensive time series of samples spanning several decades available from European cetacean stranding programmes, combining population genetics with indicators of population movements [72,73], will help test whether contemporary porpoise populations are showing a dynamic response to current climate change, and could be important in understanding how the structure of European marine ecosystems might respond to changes in the populations of such keystone predators [34].

## Supplementary Material

Electronic Supplementary Information includes 8 supplementary figures and 2 supplementary tables

## Data accessibility

All data underlying this publication are available via Dryad (http://dx.doi.org/10.5061/dryad.k4p46) [74].

## References

[1] Wiley EO 1988 Vicariance biogeography. Annu. Rev. Ecol. Syst. 19, 513–542. (doi:10.1146/annurev.es.19.110188.002501)

[2] Palumbi SR 1994 Genetic divergence, reproductive isolation, and marine speciation. Annu. Rev. Ecol. Syst. 25, 547–572. (doi:10.1146/annurev.es.25.110194.002555)

[3] Hoelzel AR 1998 Genetic structure of cetacean populations in sympatry, parapatry, and mixed assemblages: implications for conservation policy. J. Hered. 89, 451–458. (doi:10.1093/jhered/89.5.451)

[4] Fontaine MC et al. 2007 Rise of oceanographic barriers in continuous populations of a cetacean: the genetic structure of harbour porpoises in Old World waters. BMC Biol. 5, 30 (doi:10.1186/1741-7007-5-30) 17651495 10.1186/1741-7007-5-30 PMC1971045

[5] Pastene LA et al. 2007 Radiation and speciation of pelagic organisms during periods of global warming: the case of the common minke whale, *Balaenoptera acutorostrata*. Mol. Ecol. 16, 1481–1495. (doi:10.1111/j.1365-294X.2007.03244.x) 17391271 10.1111/j.1365-294X.2007.03244.x

[6] Pilot M, Dahlheim ME, Hoelzel AR 2010 Social cohesion among kin, gene flow without dispersal and the evolution of population genetic structure in the killer whale (*Orcinus orca*). J. Evol. Biol. 23, 20–31. (doi:10.1111/j.1420-9101.2009.01887.x) 19912451 10.1111/j.1420-9101.2009.01887.x

[7] Foote AD et al. 2011 Genetic differentiation among North Atlantic killer whale populations. Mol. Ecol. 21, 4854–4871. (doi:10.1111/j.1365-294X.2012.05728.x) 10.1111/j.1365-294X.2010.04957.x 21241391

[8] Louis M et al. 2014 Ecological opportunities and specializations shaped genetic divergence in a highly mobile marine top predator. Proc. R. Soc. B 281, 20141558 (doi:10.1098/rspb.1998.0416) 10.1098/rspb.2014.1558 PMC4213618 25297864

[9] Louis M et al. 2014 Habitat-driven population structure of bottlenose dolphins, *Tursiops truncatus*, in the North-East Atlantic. Mol. Ecol. 23, 857–874. (doi:10.1111/mec.12653) 24383934 10.1111/mec.12653

[10] Gaskin DE 1984 The harbour porpoise *Phocoena phocoena* (L.): regional populations, status, and information on direct and indirect catches. Rep. Int. Whal. Comm. 34, 569–586.

[11] Fontaine MC 2016 Harbour porpoises, *Phocoena phocoena*, in the Mediterranean Sea and adjacent regions: biogeographic relicts of the Last Glacial Period. Adv. Mar. Biol. 75, 333–358. (doi:10.1016/bs.amb.2016.08.006) 27770989 10.1016/bs.amb.2016.08.006

[12] Gaskin DE, Arnold PW, Blair BA 1974 Phocoena phocoena. Mamm. Species 42, 1–8. (doi:10.2307/42.1)

[13] Rosel PE, Dizon AE, Haygood MG 1995 Variability of the mitochondrial control region in populations of the harbour porpoise, *Phocoena phocoena*, on interoceanic and regional scales. Can. J. Fish. Aquat. Sci. 52, 1210–1219. (doi:10.1139/f95-118)

[14] Read AJ 1999 Harbour porpoise (*Phocoena phocoena*). In Handbook of marine mammals (eds Ridgway S, Harrison R), pp. 323–350. London, UK: Academic Press.

[15] Viaud-Martínez KA et al. 2007 Morphological and genetic differentiation of the Black Sea harbour porpoise *Phocoena phocoena*. Mar. Ecol. Prog. Ser. 338, 281–294. (doi:10.3354/meps338281)

[16] Tolley KA, Rosel PE 2006 Population structure and historical demography of eastern North Atlantic harbour porpoises inferred through mtDNA sequences. Mar. Ecol. Prog. Ser. 327, 297 (doi:10.3354/meps327297)

[17] Andersen LW, Ruzzante DE, Walton M, Berggren P, Bjørge A, Lockyer C 2001 Conservation genetics of harbour porpoises, *Phocoena phocoena*, in eastern and central North Atlantic. Conserv. Genet. 2, 309–324. (doi:10.1023/A:1012534212853)

[18] Fontaine MC et al. 2014 Postglacial climate changes and rise of three ecotypes of harbour porpoises, *Phocoena phocoena*, in western Palearctic waters. Mol. Ecol. 23, 3306–3321. (doi:10.1111/mec.12817) 24888550 10.1111/mec.12817

[19] Wiemann A et al. 2010 Mitochondrial control region and microsatellite analyses on harbour porpoise (*Phocoena phocoena*) unravel population differentiation in the Baltic Sea and adjacent waters. Conserv. Genet. 11, 195–211. (doi:10.1007/s10592-009-0023-x)

[20] Alfonsi E, Hassani S, Carpentier F-G, Le Clec'h J-Y, Dabin W, Van Canneyt O, Fontaine MC, Jung J-L 2012 A European melting pot of harbour porpoise in the French Atlantic Coasts inferred from mitochondrial and nuclear data. PLoS ONE 7, e44425 (doi:10.1371/journal.pone.0044425.t001) 22984507 10.1371/journal.pone.0044425 PMC3440431

[21] Smeenk C, Leopold MF, Addink MJ 1992 Note on the harbour porpoise *Phocoena phocoena* in Mauritania, West Africa. Lutra 35, 98–104.

[22] Donovan GP, Bjorge A 1995 Harbour porpoises in the North Atlantic: edited extract from the report of the IWC Scientific Committee, Dublin 1995. Rep. Int. Whal. Commn. S16, 3–26.

[23] Arístegui J et al. 2009 Sub-regional ecosystem variability in the Canary current upwelling. Prog. Oceanogr. 83, 33–48. (doi:10.1016/j.pocean.2009.07.031)

[24] Pinela AM, Borrell A, Cardona L, Aguilar A 2010 Stable isotope analysis reveals habitat partitioning among marine mammals off the NW African coast and unique trophic niches for two globally threatened species. Mar. Ecol. Prog. Ser. 416, 295–306. (doi:10.3354/meps08790)

[25] Pierce GJ et al. 2010 Trends in cetacean sightings along the Galician coast, north-west Spain, 2003–2007, and inferences about cetacean habitat preferences. J. Mar. Biol. Ass. 90, 1547–1560. (doi:10.1017/S0025315410000664)

[26] Méndez-Fernandez P et al. 2013 Ecological niche segregation among five toothed whale species off the NW Iberian Peninsula using ecological tracers as multi-approach. Mar. Biol. 160, 2825–2840. (doi:10.1007/s00227-013-2274-9)

[27] Santos M, Pierce G 2003 The diet of harbour porpoise (*Phocoena phocoena*) in the northeast Atlantic. Oceanogr. Mar. Biol. Annu. Rev. 41, 355–390.

[28] Spitz J, Rousseau Y, Ridoux V 2006 Diet overlap between harbour porpoise and bottlenose dolphin: an argument in favour of interference competition for food? Estuar. Coast Shelf Sci. 70, 259–270. (doi:10.1016/j.ecss.2006.04.020)

[29] Moritz C 2002 Strategies to protect biological diversity and the evolutionary processes that sustain it. Syst. Biol. 51, 238–254. (doi:10.1080/10635150252899752) 12028731 10.1080/10635150252899752

[30] Fontaine MC, Snirc A, Frantzis A, Koutrakis E, Öztürk B, Oztürk AA, Austerlitz F 2012 History of expansion and anthropogenic collapse in a top marine predator of the Black Sea estimated from genetic data. Proc. Natl Acad. Sci. USA 109, E2569–E2576. (doi:10.1073/pnas.1201258109) 22949646 10.1073/pnas.1201258109 PMC3458354

[31] Fontaine MC et al. 2010 Genetic and historic evidence for climate-driven population fragmentation in a top cetacean predator: the harbour porpoises in European waters. Proc. R. Soc. B 277, 2829–2837. (doi:10.1098/rspb.2010.0412) 10.1098/rspb.2010.0412 PMC2981983 20444724

[32] Wright S 1943 Isolation by Distance. Genetics 28, 114–138. 17247074 10.1093/genetics/28.2.114 PMC1209196

[33] Rousset F 1997 Genetic differentiation and estimation of gene flow from F-statistics under isolation by distance. Genetics 145, 1219–1228. 9093870 10.1093/genetics/145.4.1219 PMC1207888

[34] Beaugrand G, Edwards M, Raybaud V, Goberville E, Kirby RR 2015 Future vulnerability of marine biodiversity compared with contemporary and past changes. Nat. Clim. Change 5, 695–701. (doi:10.1038/nclimate2650)

[35] Jepson PD 2003 Pathology and toxicology of stranded harbour porpoises (*Phocoena phocoena*) in UK waters. PhD thesis, University of London, London, UK.

[36] Law RJ 1994 Collaborative UK marine mammal project: summary of data produced 1988–1992. Fisheries Research Technical Report. Lowestoft, UK: MAFF Directorate of Fisheries Research.

[37] R Core Team. 2016 R: a language and environment for statistical computing. Vienna, Austria: R Foundation for Statistical Computing.

[38] Pante E, Simon-Bouhet B 2013 marmap: a package for importing, plotting and analyzing bathymetric and topographic data in R. PLoS ONE 8, e73051 (doi:10.1371/journal.pone.0073051) 24019892 10.1371/journal.pone.0073051 PMC3760912

[39] NOAA-NGDC. *2-Minute Gridded Global Relief Data (ETOPO2v2)*. See http://www.ngdc.noaa.gov/mgg/fliers/06mgg01.html (accessed 27 November 2016).

[40] Jepson PD (ed) 2005 Cetacean strandings investigation and co-ordination in the UK 2000–2004. Final report to the Department for Environment, Food and Rural Affairs, pp. 1–79.

[41] Jepson PD, Perkins M, Brownlow A, Davinson NJ, Doeschate ten M, Smith B, Lyal R, Sabin R, Penrose R 2014 Annual Report for the period 1 January–31 December 2014 (contract number MB0111), pp. 1–62.

[42] NODC-NOAA. World Ocean Atlast 01. See http://www.nodc.noaa.gov/OC5/WOA01/qd_ts01.html (accessed 27 November 2016).

[43] NASA. Sea-viewing wide field-of-view sensor database (SeaWIFS). See http://oceancolor.gsfc.nasa.gov/cms/ (accessed 27 November 2016).

[44] Kamvar ZN, Tabima JF, Grünwald NJ 2014 Poppr: an R package for genetic analysis of populations with clonal, partially clonal, and/or sexual reproduction. PeerJ 2, e281 (doi:10.7717/peerj.281/table-6) 24688859 10.7717/peerj.281 PMC3961149

[45] Weir BS, Cockerham CC 1984 Estimating F-statistics for the analysis of population structure. Evolution 38, 1358–1370. (doi:10.2307/2408641) 10.1111/j.1558-5646.1984.tb05657.x 28563791

[46] Belkhir K, Borsa P, Chikhi L, Raufaste N, Bonhomme F 2004 GENETIX 4.05, logiciel sous Windows TM pour la génétique des populations. Laboratoire Génome, Populations, Interactions, CNRS UMR 5171, Université de Montpellier II, Montpellier, France.

[47] Goudet J 2001 FSTAT, a program to estimate and test gene diversities and fixation indices (version 2.9. 3). See http://www2.unil.ch/popgen/softwares/fstat.htm (accessed 8 September 2016).

[48] Keenan K, McGinnity P, Cross TF, Crozier WW, Prodöhl PA 2013 DiveRsity: an R package for the estimation and exploration of population genetics parameters and their associated errors. Methods Ecol. Evol. 4, 782–788. (doi:10.1111/2041-210X.12067)

[49] Szpiech ZA, Jakobsson M, Rosenberg NA 2008 ADZE: a rarefaction approach for counting alleles private to combinations of populations. Bioinformatics 24, 2498–2504. (doi:10.1093/bioinformatics/btn478) 18779233 10.1093/bioinformatics/btn478 PMC2732282

[50] Pebesma EJ 2004 Multivariable geostatistics in S: the gstat package. Comput. Geosci. 30, 683–691. (doi:10.1016/j.cageo.2004.03.012)

[51] Falush D, Stephens M, Pritchard JK 2003 Inference of population structure using multilocus genotype data: linked loci and correlated allele frequencies. Genetics 164, 1567–1587. 12930761 10.1093/genetics/164.4.1567 PMC1462648

[52] Pritchard JK, Stephens M, Donnelly P 2000 Inference of population structure using multilocus genotype data. Genetics 155, 945–959. 10835412 10.1093/genetics/155.2.945 PMC1461096

[53] Hubisz MJ, Falush D, Stephens M, Pritchard JK 2009 Inferring weak population structure with the assistance of sample group information. Mol. Ecol. Resour. 9, 1322–1332. (doi:10.1111/j.1755-0998.2009.02591.x) 21564903 10.1111/j.1755-0998.2009.02591.x PMC3518025

[54] Kopelman NM, Mayzel J, Jakobsson M, Rosenberg NA, Mayrose I 2015 Clumpak: a program for identifying clustering modes and packaging population structure inferences across K. Mol. Ecol. Resour. 15, 1179–1191. (doi:10.1111/1755-0998.12387) 25684545 10.1111/1755-0998.12387 PMC4534335

[55] Evanno G, Regnaut S, Goudet J 2005 Detecting the number of clusters of individuals using the software structure: a simulation study. Mol. Ecol. 14, 2611–2620. (doi:10.1111/j.1365-294X.2005.02553.x) 15969739 10.1111/j.1365-294X.2005.02553.x

[56] Fontaine MC, Galan M, Bouquegneau J-M, Michaux JR 2006 Efficiency of fluorescent multiplex polymerase chain reactions (PCRs) for rapid genotyping of harbour porpoises (*Phocoena phocoena*) with 11 microsatellite loci. Aqua. Mamm. 32, 301–304. (doi:10.1578/AM.32.3.2006.301)

[57] Jombart T, Pontier D, Dufour A-B 2009 Genetic markers in the playground of multivariate analysis. Heredity 102, 330–341. (doi:10.1038/hdy.2008.130) 19156164 10.1038/hdy.2008.130

[58] Mcvean G 2009 A genealogical interpretation of principal components analysis. PLoS Genet. 5, e1000686 (doi:10.1371/journal.pgen.1000686.g006) 19834557 10.1371/journal.pgen.1000686 PMC2757795

[59] Francois O, Durand E 2010 Spatially explicit Bayesian clustering models in population genetics. Mol. Ecol. Resour. 10, 773–784. (doi:10.1111/j.1755-0998.2010.02868.x) 21565089 10.1111/j.1755-0998.2010.02868.x

[60] Jombart T, Devillard S, Dufour A-B, Pontier D 2008 Revealing cryptic spatial patterns in genetic variability by a new multivariate method. Heredity 101, 92–103. (doi:10.1038/hdy.2008.34) 18446182 10.1038/hdy.2008.34

[61] Jombart T, Ahmed I 2011 *Adegenet* 1.3-1: new tools for the analysis of genome-wide SNP data. Bioinformatics 27, 3070–3071. (doi:10.1093/bioinformatics/btr521) 21926124 10.1093/bioinformatics/btr521 PMC3198581

[62] Rousset F 2000 Genetic differentiation between individuals. J. Evol. Biol. 13, 58–62. (doi:10.1046/j.1420-9101.2000.00137.x)

[63] Rousset F 2004 Genetic structure and selection in subdivided populations. Princeton, NJ: Princeton University Press.

[64] Hardy OJ, Vekemans X 2002 Spagedi: a versatile computer program to analyse spatial genetic structure at the individual or population levels. Mol. Ecol. Notes 2, 618–620. (doi:10.1046/j.1471-8278.2002.00305.x)

[65] Ray N 2005 pathmatrix: a geographical information system tool to compute effective distances among samples. Mol. Ecol. Notes 5, 177–180. (doi:10.1111/j.1471-8286.2004.00843.x)

[66] Dinter W 2001 Biogeography of the OSPAR maritime area. Bonn, Germany: Federal Agency for Nature Conservation.

[67] OSPAR. 2010 Quality Status Report 2010. 2 The North-East Atlantic. In Quality Status Report 2010, pp. 1–176. London, UK: OSPAR Commission.

[68] Wang JY, Berggren P 1997 Mitochondrial DNA analysis of harbour porpoises (*Phocoena phocoena*) in the Baltic Sea, the Kattegat-Skagerrak Seas and off the west coast of Norway. Mar. Biol. 127, 531–537. (doi:10.1007/s002270050042)

[69] Rosel PE, France SC, Wang JY, Kocher TD 1999 Genetic structure of harbour porpoise *Phocoena phocoena* populations in the northwest Atlantic based on mitochondrial and nuclear markers. Mol. Ecol. 8, S41–S54. (doi:10.1046/j.1365-294X.1999.00758.x) 10703550 10.1046/j.1365-294x.1999.00758.x

[70] Siebert U, Gilles A, Lucke K, Ludwig M, Benke H, Kock K-H, Scheidat M 2006 A decade of harbour porpoise occurrence in German waters—analyses of aerial surveys, incidental sightings and strandings. J. Sea Res. 56, 65–80. (doi:10.1016/j.seares.2006.01.003)

[71] Verfuß UK, Honnef CG, Meding A, Dähne M, Mundry R, Benke H 2007 Geographical and seasonal variation of harbour porpoise (*Phocoena phocoena*) presence in the German Baltic Sea revealed by passive acoustic monitoring. J. Mar. Biol. Ass. 87, 165 (doi:10.1017/S0025315407054938)

[72] Peltier H et al. 2013 The stranding anomaly as population indicator: the case of harbour porpoise *Phocoena phocoena* in North-Western Europe. PLoS ONE 8, e62180 (doi:10.1371/journal.pone.0062180) 23614031 10.1371/journal.pone.0062180 PMC3632559

[73] Hammond PS et al. 2013 Cetacean abundance and distribution in European Atlantic shelf waters to inform conservation and management. Biol. Conserv. 164, 107–122. (doi:10.1016/j.biocon.2013.04.010)

[74] Fontaine MC, Thatcher O, Ray N, Piry S, Brownlow A, Davison NJ, Jepson P, Deaville R, Goodman SJ 2017 Data from: Mixing of porpoise ecotypes in southwestern UK waters revealed by genetic profiling. Dryad Digital Repository. (http://dx.doi.org/10.5061/dryad.k4p46) 10.1098/rsos.160992 PMC5383846 28405389

