# Supplementary material for: Mixing of porpoise ecotypes in southwestern UK waters revealed by genetic profiling
Source: R Soc Open Sci. 2017 Mar 1;4(3):160992. doi: 10.1098/rsos.160992 (PMC5383846; doi:10.1098/rsos.160992)
Supplement: Electronic Supplementary Information includes 8 supplementary figures and 2 supplementary tables [file rsos160992supp1.pdf]

Royal Society Open Science
Electronic Supplementary Information

Mixing of Porpoise Ecotypes in South Western UK Waters Revealed by Genetic Profiling

Michaël C. Fontaine, Oliver Thatcher, Nicolas Ray, Sylvain Piry, Andrew Brownlow, Nicholas J. Davison, Paul Jepson, Rob Deaville, Simon J. Goodman

Table S1. Microsatellite loci used in this study.

Table S2. Geographic differences in residuals from the model [Ln(lenght) ~ Ln(age) + sex] between all pairs of regions. The value reported are the *P-values* of Tukey multiple comparison test.

Figure S1. Geographic distribution of the sampling of harbour porpoise stratified by age class or by sex.

Figure S2. Geographic distribution of the sampling stratified by sex and by age-class (juveniles and adults).

Figure S3. Geographic distribution of the sampling stratified by year.

Figure S4. Count distribution of the sampling across the years stratified by age classes.

Figure S5. Proportion of missing data per locus and per geographic group.

Figure S6. (*a*) Posterior probability of *K* as estimated in *Structure* using Pritchard approach (see Pritchard *et al.* 2000 and *Structure* v.2.3.4 user manual) and (*b*) Evanno's Delta-K (Evanno *et al.* 2005). (*c*) Geographic distribution of the parental *pure* populations from the southern and northern ecotype of harbour porpoise and the hybrid. Pure individuals were identified with *Structure* analysis as individuals having their multilocus genotypes assigned with > 80% probability to their respective cluster or as hybrids if the probability was < 80% (see figure 2). Only the Iberian (IB) population from the southern ecotype is shown. Hybrids (HYB) porpoises are geographically restricted to the northern side of the Bay of Biscay, Celtic Sea, and SW UK, with some individuals found in the western side of the channel and in Scotland.

Figure S7. (*a*) Geographic locations of the harbour porpoises sampling (n=591) based on GPS coordinates or reported discovery location. Locations have been subdivided into 6 regions around UK and color-coded accordingly. Genetic structure of harbour porpoises in UK waters at K=2, as estimated by *Structure*, is displayed as the posterior admixture estimates averaged per regions. Panel (*b*) shows the individual admixture proportions. Each individual is represented by a column and the probability of that individual belonging to each cluster is indicated by coloured segments. Admixture proportions from *Structure* are based on the highest probability run (of ten) at that value of K=2.

Figure S8. Allelic richness estimated for a minimum sample size of n=2 and its variance using ADZE. Spatially interpolated surfaces were computed using an inverse distance weighted method on a gridded space of 1°. Red circle show each sample location and the black dots show the pixel where n ≥ 2 for which Ra was estimated on the local sampling.

Table S1. Microsatellite loci used in this study.

Locus	Ref.	Primer-F	Ta (ºC)	Repeat nº
**Igf-1a**	a,b	F: GGGTATTGCTAGCCAGCTGGT<br>R: CATATTTTTCTGCATAACTTGAACCT	52	2
**415/416**	b,c	F: GTTCCTTTCCTTACA<br>R: ATCAATGTTTGTCAA	40	2
**417/418**	b,c	F: GTGATATCATACAGTA<br>R: ATCTGTTTGTCACATA	46	2
**GT011**	d	F: CATTTTGGGTTGGATCATTC<br>R: GTGGAGACCAGGGATATTGC	59	2
**GT015**	e	F: GAGAATGGCTGGGCTCAGATC<br>R: TTCCCTATTAGAGGCTCACGA	59	2
**GT101**	e	F: AGAATGGAGTTGTGTAACAGG<br>R: CTTTCTCCTAGTGCTCCCCGC	56	2
**GT136**	e	F: AAAAAGTCTCCTCTGGACCTG<br>R: GTGCACCCTGGACTGTTAGTG	52	2
**EV104**	e	F: TGGAGATGACAGGATTTGGG<br>R: GGAATTTTTATTGTAATGGGTCC	48/52	2
**EV94**	f	F: ATCGTATTGGTCCTTTTCTGC<br>R: AATAGATAGTGATGATGATTCACACC	48/56	2
**EV96**	f	F: AAGATGAGTAGATTCACTACACGAG<br>R: GCCACTTTTCCTCTCACATAGCC	48/56	2
**TAA031**	g	F: TCCAGTGGTTAGGACTTGGCG<br>R: TCACTTCCTACTTTGATGAGG	53	3
**GATA053**	g	F: ATTGGCAGTGGCAGGAGACCC<br>R: GGTGAGTGAGTGATGCAGAGG	55	4

a, Kirkpatrick (1992); b, Andersen et al. (1997); c, Amos et al. (1993); d, Bérubé et al., (1998); e, Andersen et al. (2001); f, Valsecchi & Amos (1996); g, Palsbøll et al. (1997).

Table S2. Geographic differences in residuals from the model [Ln(lenght) ~ Ln(age) + sex] between all pairs of regions. The value reported are the *P-values* of Tukey multiple comparison test.

	Channel	Cwest	NSN	NSS	West	Wscot
**Channel**	-	< 0.001	1.000	0.981	0.432	0.995
**Cwest**		-	< 0.001	< 0.001	< 0.001	< 0.001
**NSN**			-	0.768	0.002	0.972
**NSS**				-	0.114	1.000
**West**					-	0.174
**Wscot**						-

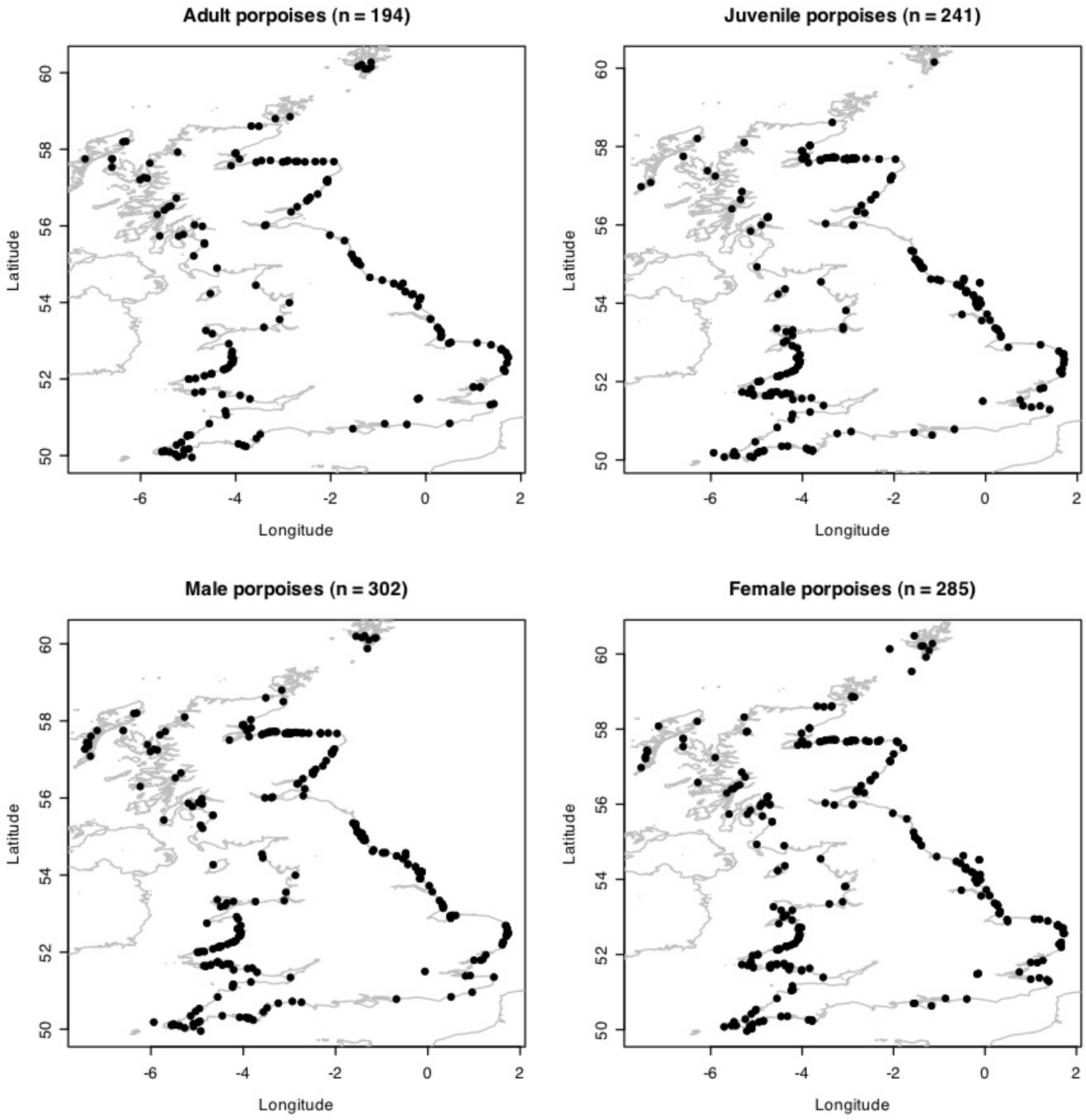

Figure S1. Geographic distribution of the sampling of harbour porpoise stratified by age class or by sex.

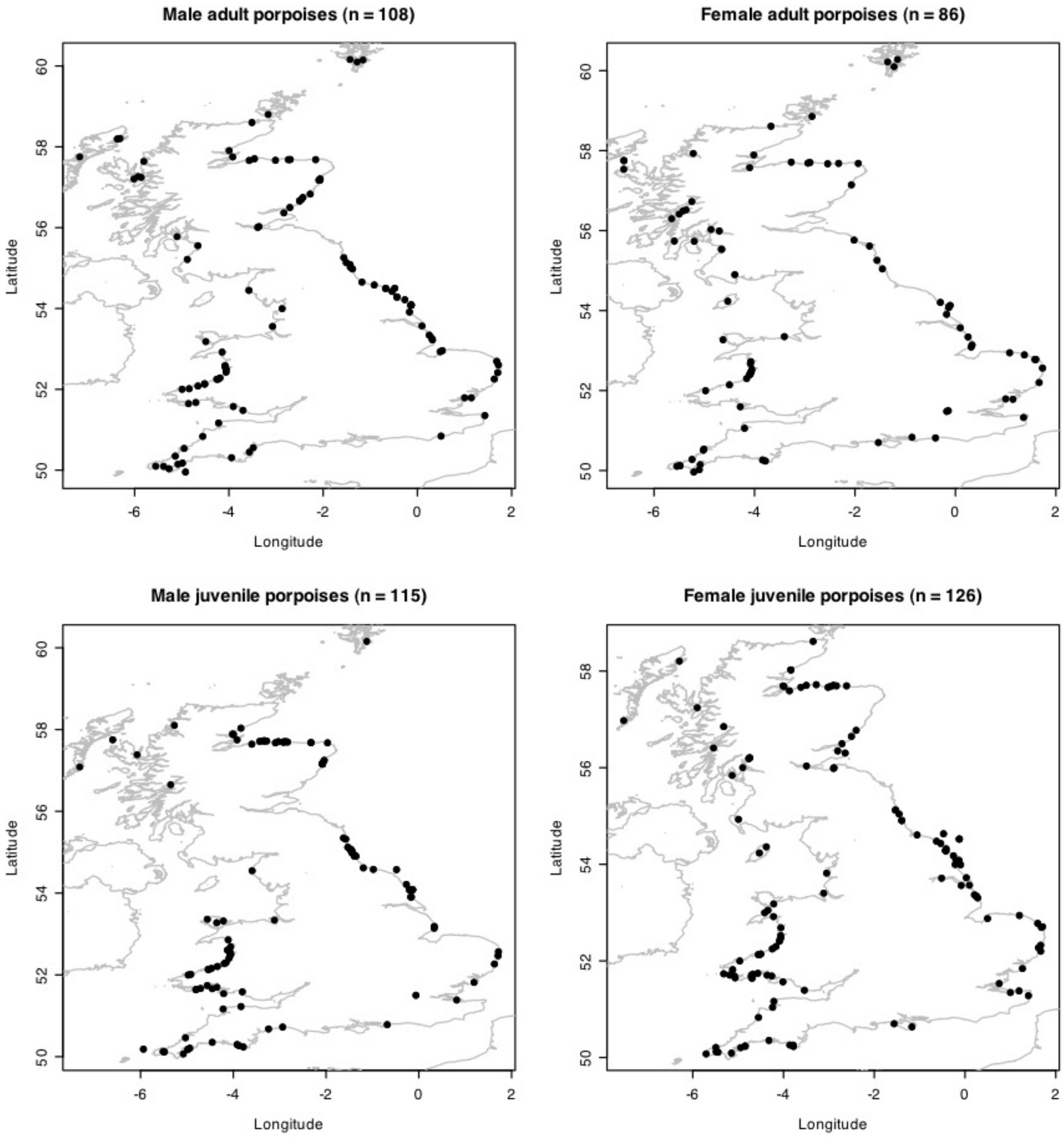

Figure S2. Geographic distribution of the sampling stratified by sex and by age-class (juveniles and adults).

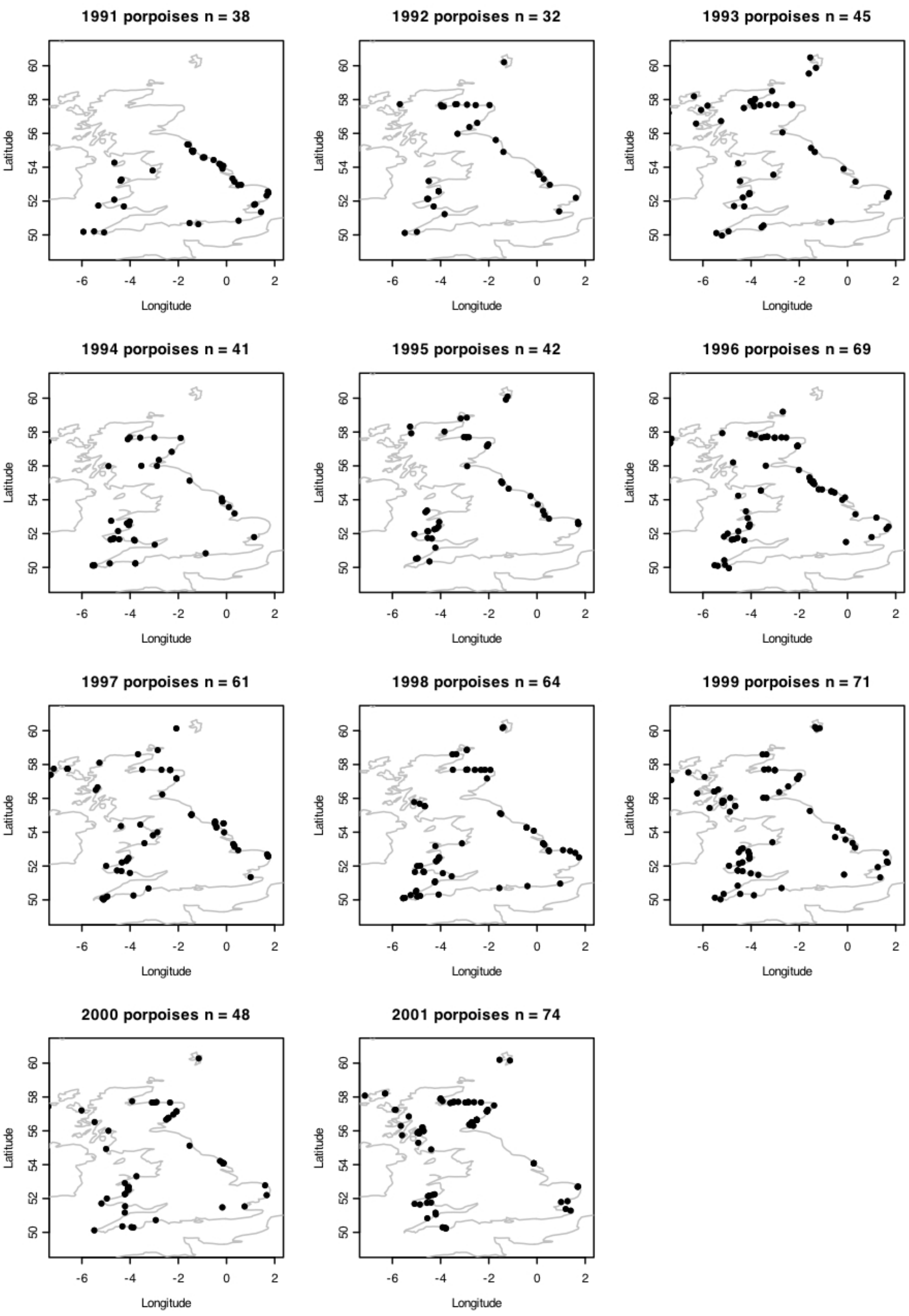

Figure S3. Geographic distribution of the sampling stratified by year.

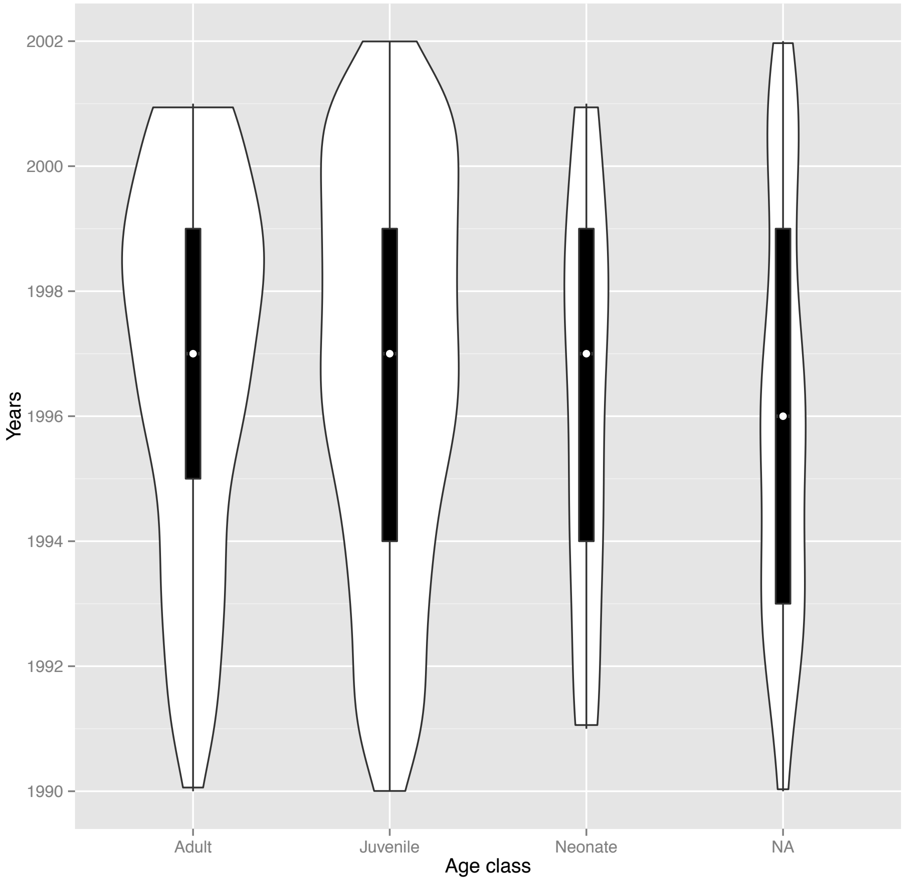

Figure S4. Count distribution of the sampling across the years stratified by age classes.

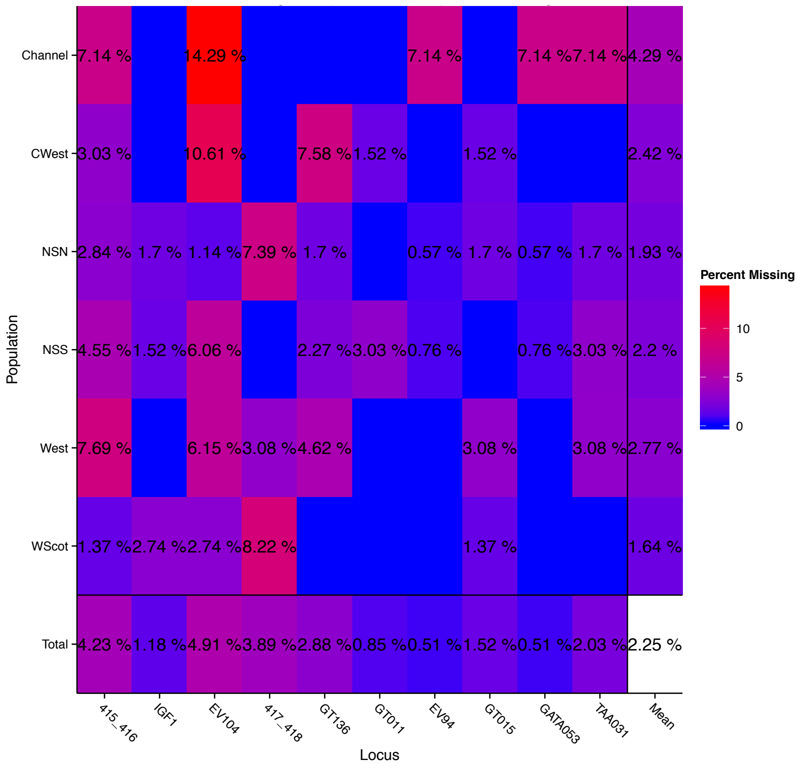

Figure S5. Proportion of missing data per locus and per geographic group.

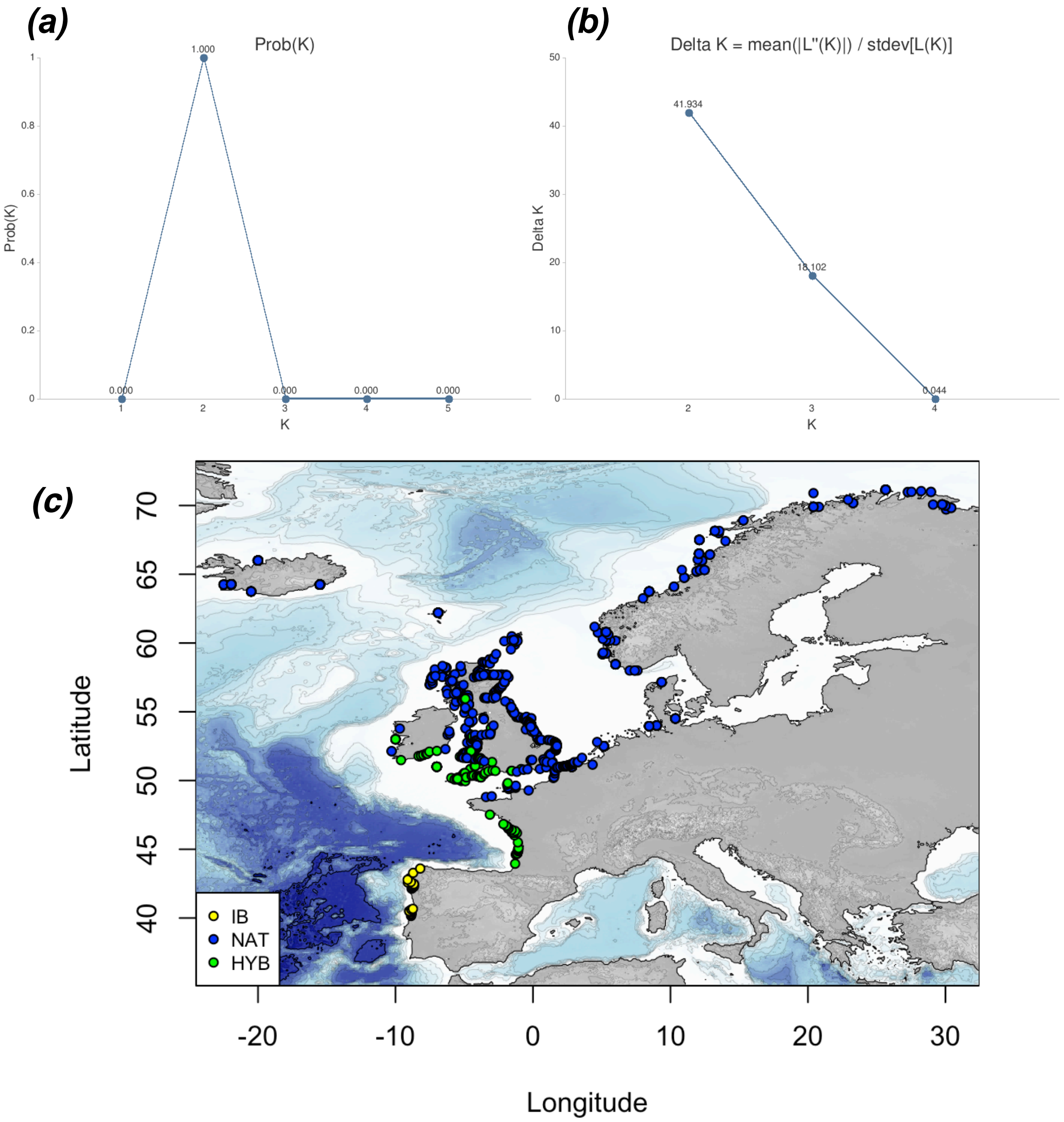

Figure S6. (*a*) Posterior probability of *K* as estimated in *Structure* using Pritchard approach (see Pritchard *et al.* 2000 and *Structure* v.2.3.4 user manual) and (*b*) Evanno's Delta-K (Evanno *et al.* 2005). (*c*) Geographic distribution of the parental *pure* populations from the southern and northern ecotype of harbour porpoise and the hybrid. Pure individuals were identified with *Structure* analysis as individuals having their multilocus genotypes assigned with > 80% probability to their respective cluster or as hybrids if the probability was < 80% (see figure 2). Only the Iberian (IB) population from the southern ecotype is shown. Hybrids (HYB) porpoises are geographically restricted to the northern side of the Bay of Biscay, Celtic Sea, and SW UK, with some individuals found in the western side of the channel and in Scotland.

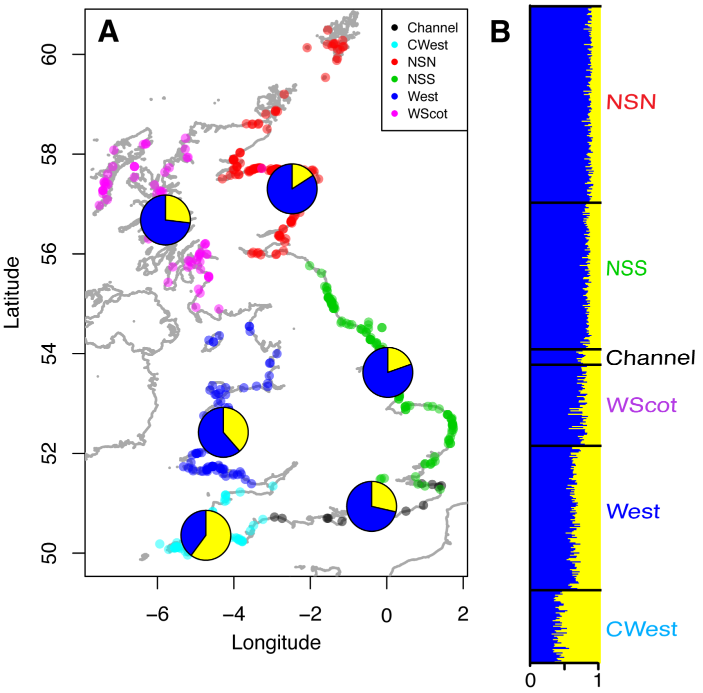

Figure S7. (*a*) Geographic locations of the harbour porpoises sampling (n=591) based on GPS coordinates or reported discovery location. Locations have been subdivided into 6 regions around UK and color-coded accordingly. Genetic structure of harbour porpoises in UK waters at K=2, as estimated by *Structure*, is displayed as the posterior admixture estimates averaged per regions. Panel (*b*) shows the individual admixture proportions. Each individual is represented by a column and the probability of that individual belonging to each cluster is indicated by coloured segments. Admixture proportions from *Structure* are based on the highest probability run (of ten) at that value of K=2.

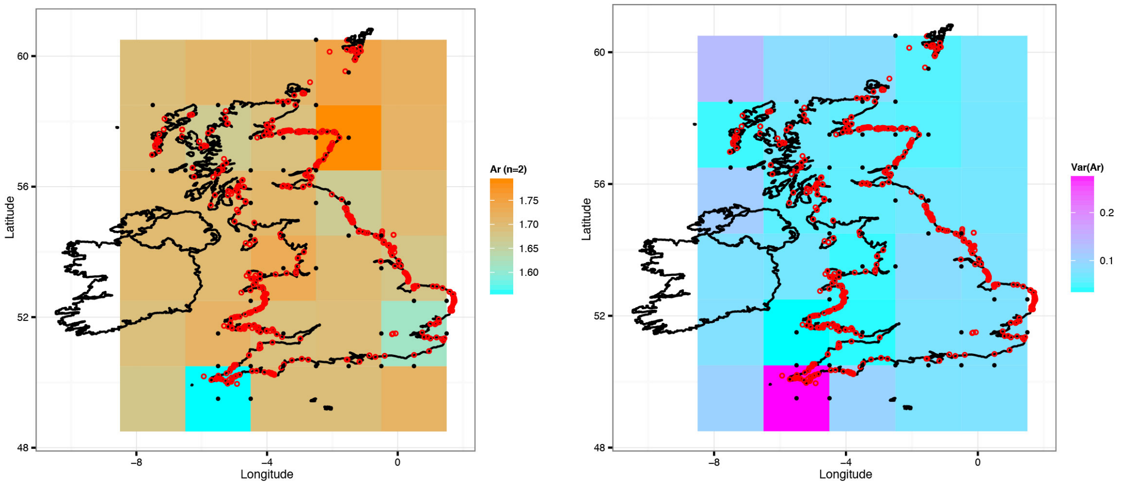

Figure S8. Allelic richness estimated for a minimum sample size of n=2 and its variance using ADZE. Spatially interpolated surface were computed using an inverse distance weighted method on a gridded space of 1º. Red circle show each sample location and the black dots show the pixel where $n \geq 2$ for which Ra was estimated on the local sampling.
